# ETM-DB: integrated Ethiopian traditional herbal medicine and phytochemicals database

**DOI:** 10.1186/s12906-019-2634-1

**Published:** 2019-08-14

**Authors:** Lemessa Etana Bultum, Assefa Mussa Woyessa, Doheon Lee

**Affiliations:** 1 Department of Bio and Brain Engineering, Korea Advanced Institute of Science and Technology, Daejeon, 34141 South Korea; 2 Bio-Synergy Research Center, Daejeon, 34141 South Korea

**Keywords:** Ethiopia, Integrated database, Traditional medicine, Phytochemicals

## Abstract

**Background:**

Recently, there has been an increasing tendency to go back to nature in search of new medicines. To facilitate this, a great deal of effort has been made to compile information on natural products worldwide, and as a result, many ethnic-based traditional medicine databases have been developed. In Ethiopia, there are more than 80 ethnic groups, each having their indigenous knowledge on the use of traditional medicine. About 80% of the population uses traditional medicine for primary health care. Despite this, there is no structured online database for Ethiopian traditional medicine, which limits natural products based drug discovery researches using natural products from this country.

**Description:**

To develop ETM-DB, online research articles, theses, books, and public databases containing Ethiopian herbal medicine and phytochemicals information were searched. These resources were thoroughly inspected and the necessary data were extracted. Then, we developed a comprehensive online relational database which contains information on 1054 Ethiopian medicinal herbs with 1465 traditional therapeutic uses, 573 multi-herb prescriptions, 4285 compounds, 11,621 human target gene/proteins, covering 5779 herb-phenotype, 1879 prescription-herb, 16,426 herb-compound, 105,202 compound-phenotype, 162,632 compound-gene/protein, and 16,584 phenotype-gene/protein relationships. Using various cheminformatics tools, we obtained predicted physicochemical and absorption, distribution, metabolism, excretion, and toxicity (ADMET) properties of ETM-DB compounds. We also evaluated drug-likeness properties of these compounds using FAF-Drugs4 webserver. From the 4285 compounds, 4080 of them passed the FAF-Drugs4 input data curation stage, of which 876 were found to have acceptable drug-likeness properties.

**Conclusion:**

ETM-DB is the largest, freely accessible, web-based integrated resource on Ethiopian traditional medicine. It provides traditional herbal medicine entities and their relationships in well-structured forms including reference to the sources. The ETM-DB website interface allows users to search the entities using various options provided by the search menu. We hope that our database will expedite drug discovery and development researches from Ethiopian natural products as it contains information on the chemical composition and related human target gene/proteins. The current version of ETM-DB is openly accessible at http://biosoft.kaist.ac.kr/etm.

## Background

Natural products (NPs) and natural product structures continue to play a significant role in the drug discovery and development process. Specifically, secondary metabolites of plants produced for defense have medicinal value [1]. An analysis of the number and sources of drugs newly approved by the US Food and Drug Administration (FDA) between 1981 and 2014 revealed that more than 50% of these drugs were natural product based, including 65% of anticancer, 48% of antidiabetic, and 36% of antiviral medicines [2]. Recently, the use of natural products as traditional, complementary and alternative medicine (TCAM) is increasing in developed countries. In the United States, complementary and alternative medicine practice has nearly doubled in popularity and acceptance in the last decade [3]. Ethiopia is home to many ethnic groups and cultures, which in turn have contributed to the high diversity of traditional health care knowledge and practices. For centuries, the people heavily relied on traditional medicine to treat various physical and mental disorders. It is estimated that about 80% of the Ethiopian population, predominantly in rural areas, use traditional medicine due to its accessibility and affordability [4, 5]. The flora of Ethiopia is estimated to have between 6500 and 7000 species, of which 10–12% is considered endemic [6]. Around 1000 plant species, more than 15% of the total flora, are reported to have medicinal value [7]. It is also believed that there are still multitudes of unidentified medicinal plant species in Ethiopia. Recently, there has been an encouraging development in the country to promote the wide-scale use and commercialization of some of these species.

There are many traditional medicine databases worldwide covering diverse and valuable information for researchers in drug discovery and development. Databases produced upon western and eastern natural products have different emphases. Eastern databases tend to place more emphasis on herbal ingredients and therapeutic effects, while most western databases are compound based and focus more on mechanisms than origin or folk use [8]. Some of the well-known eastern natural product databases include Traditional Chinese Medicine (TCM) Integrative Database (TCMID) [9], TCM Information Database (TCM-ID) [10], TCM Database@Taiwan [11], Chinese Ethnic Minority Traditional Drug Database (CEMTDD) [12], A Comprehensive Species-Metabolite Relationship Database (KNApSAcK) [13], A curated database of Indian Medicinal Plants, Phytochemistry And Therapeutics (IMPPAT) [14], and Phytochemica [15]. Some examples of western natural product databases are Natural Products Alert (NAPRALERT) [16], Nutrichem [17], and Super Natural [18]. These databases provide information on various aspects of natural products such as medicinal herbs, compounds, target gene/proteins, diseases, metabolic toxicity, and so on.

There have been few studies on African natural products, which led to the development of some national and regional databases. The notable ones are AfroDB [19], ConMedNP [20], CamMedNP [21], p-ANAPL [22], South African natural compound database (SANCDB) [23], and the most recently published - A Resource for Natural Products from Northern African Sources (NANPDB) [24]. The latter contains about 4500 compounds, which were identified from northern African natural resources in literatures between 1962 and 2016. The SANCDB contains chemical structural data of 600 compounds derived from 143 different South African organisms. The CamMedNP and ConMedNP libraries contain three-dimensional (3D) models of compounds from the Central African region. AfroDb includes a data set of about 1000 compounds obtained from natural resources of various African regions. The Pan-African Natural Products Library (p-ANAPL) is composed of about 600 compounds from some African plants. In general, these databases focus on compounds isolated from central, western, southern, and northern African natural resources.

There has also been an attempt to compile natural products information from Ethiopia and East Africa, notably the Natural Database for Africa (NDA) [25]. This database covers information on 7000 plant species, mainly from Ethiopia and some from neighbouring African countries. It mostly contains herb information, such as botanical names, vernacular names, and traditional uses. However, it does not contain information on the constituting compounds and target gene/proteins, which is important for drug discovery researches. Furthermore, even the available information such as traditional therapeutic uses are not properly structured. Therefore, Ethiopian traditional medicine knowledge is still scattered in different literature sources ranging from modern research articles to centuries old traditional literatures. The modern literatures (e.g., research reports, surveys, and books) come from academic fields such as ethnobotany, ethnomedicine, phytochemical studies, and the biological evaluation of plants. Traditional literatures are based on oral testimonies and old manuscripts written by local traditional medicine practitioners. Some examples of these traditional literatures are medico-religious manuscripts inscribed in parchments written in Geez, liturgical language of the Ethiopian church, in the fifteenth century and the Book of Remedy (Metsehafe Fews- ) from the seventeenth century, which contains a wide range of herbal medicine prescriptions [4, 7].

It is important to note that very few research articles appear in peer-reviewed journals from Ethiopia. Many of the researches are reported in low-level publications or as higher degree theses. Although these resources contain valuable knowledge, it has been difficult for researchers to find. The scattered, unstructured, and non-electronic nature of data on Ethiopian traditional medicine restricts their wide utilization in drug discovery. Therefore, consolidation of these data into a database can assist researchers to find well-structured information on Ethiopian traditional medicine.

## Construction and content

### Data collection and assembly

Ethiopian traditional medicines information is scattered across different sources. Therefore, the first step in developing the ETM-DB was to collect and document research articles, theses, and books containing relevant data on Ethiopian herbal medicines and phytochemicals. The research articles were mainly searched from open source publishers such as Journal of Ethnopharmacology, Ethnobiology and Ethnomedicine, Pharmaceutical and Medical Research, Phytotherapy Research, Science & Development, Plant Science, Medicinal Plant Research, Pharmacy and Chemistry, Pharmaceuticals and Health Care Research, Medicinal Plants Studies, African Botany, Biodiversity and Conservation, Evidence-Based Complementary and Alternative Medicine, and Biological Sciences. In total, 48 research articles, 10 theses, 3 books, and 5 public databases involving natural products were used to collect the data for ETM-DB construction. The books we used were African Herbal Pharmacopeia [26], Handbook of African Medicinal Plants [27], and Medicinal plants and Enigmatic Health Practices of Northern Ethiopia [28]. Two of the books, African Herbal Pharmacopeia and Handbook of African Medicinal Plants, contain a geographical distribution of the herbs and we manually extracted data on Ethiopian herbs. The theses and articles focus on ethnobotanical studies and mainly contain herbs and symptoms information. The books contain information on herbs, prescriptions, symptoms, and related compounds. The databases contain herbs, symptoms, related compounds, and target gene/proteins information. Generally, the literature search and manual data collection resulted in 1054 distinct herbs with their therapeutic use and some herb-compound relationships.

After manual data collection, we used public databases to standardize the herb (The Plant List [29]), to collect herb synonyms and National Center for Biotechnology Information Identifier (NCBI ID) (NCBI Taxonomy [30]), to obtain more herb related compounds (Compound Combination-Oriented Natural Product Database with Unified Terminology (COCONUT) [31]), and to get attributes and properties for the compounds (absorption, distribution, metabolism, excretion, and toxicity (ADMET) structure-activity relationship database (admetSAR) [32], PubChem [33], and ChemSpider [34]). The data from these databases were extracted using either python or java codes.

**Fig. 1:**
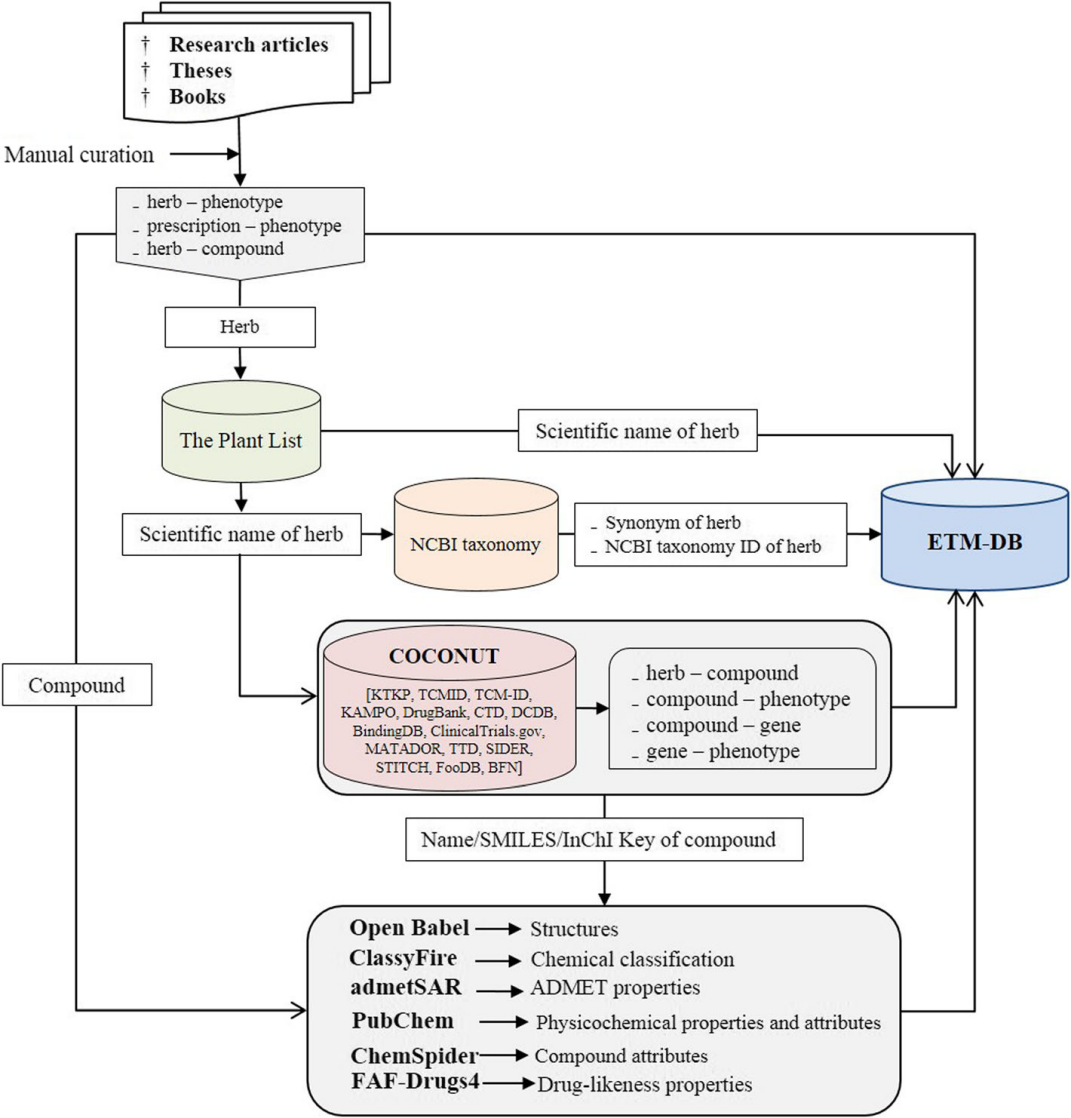
Illustration of ETM-DB development process. Concisely, we compiled herb-phenotype, prescription-herb, prescription-phenotype, herb-compound, compound-phenotype, compound-gene/protein, and phenotype-gene/protein associations of Ethiopian traditional medicine from literatures and database sources. We organized the information into a relational database. In addition, we used cheminformatics methods to obtain chemical structure, chemical classes, predicted physicochemical, and ADMET properties of the compounds. We also evaluated the drug-likeness of the compounds using the physicochemical properties

The reference to data sources is included in each record. For herbs, compounds, and gene/proteins, representative international identifiers were included so that users can access further information. This will enhance the compatibility of ETM-DB with other databases. The brief overview of ETM-DB development process is illustrated in Fig. 1.

#### Ethiopian traditional herbal medicine

At the beginning of ETM-DB development, medicinal herbs and related information were manually curated from research articles, theses, and books resulting in identification and documentation of 1054 distinct herbs. Since the herbs name varies across literatures and geographical regions, we manually checked and standardized the herbs’ common names to their corresponding scientific names using The Plant List database. Using their scientific names, we checked the herbs’ availability in public databases, namely NCBI taxonomy and COCONUT. We found that 543 herbs are available in NCBI taxonomy while 218 herbs are available in COCONUT. All of the 218 herbs in COCONUT overlap with the herbs available in NCBI taxonomy. We have included NCBI taxonomy ID and synonyms for the 543 herbs in ETM-DB.

We also manually compiled therapeutic use of the herbs. It includes phenotype symptoms the herb is used to treat, parts of the herb in usage, and how to use it. Then, we standardized the symptoms using MetaMap [35], a text-mining software which maps narrative text to concepts in the Unified Medical Language System (UMLS) [36] through the named entity recognition (NER) method. Hence, the UMLS concept identifier and UMLS concept name are included as attributes of the phenotype entity. This is the first effort to map ethnopharmacological information on Ethiopian traditional medicine with standardized terminology in contemporary medicine.

In addition to the therapeutic uses of individual herbs, we compiled multi-herb prescriptions from the book Medicinal plants and Enigmatic Health Practices of Northern Ethiopia. This accumulated to 573 multi-herb prescription information from some combination of 265 herbs.

#### Compound composition of Ethiopian medicinal herbs

Herbs are widely used in traditional medicine. Identifying the essential compounds constituting the herbs are crucial to utilize them in modern drug discovery and development researches. Currently, we have compiled 4285 distinct compounds found in Ethiopian medicinal herbs. We used the Simplified Molecular Input Line Entry System (SMILES) string to identify unique compounds and avoid redundancies arising from synonymic names. Generally, we have collected 1024 herb-compound relationships from the books manually and 15,402 herb-compound relationships from the COCONUT database using python script.

#### Compound attributes and general properties

>We used ChemSpider and PubChem databases to standardize, annotate, and provide general properties of ETM-DB compounds. ChemSpider is a free online chemical structure resource database, which provides various information about compounds that can be searched either by chemical names or by other identifiers such as SMILES, International Chemical Identifier (InChI), InChIKey, etc. PubChem is a public repository for information on chemical substances and their biological activities. We used Java programming language to implement ChemSpider’s web service search SOAP 1.2 using compound names as input to extract ChemSpider identifier, SMILES, InChI, and InChIKey. We used PubChemPy, a python library that can interact with PubChem database, to get PubChem identifier, synonyms, two-dimensional (2D) structural depictions (PNG image files), SMILES, InChI, InChIKey, and computed physicochemical properties of the compounds. 2D and 3D structure data files (SDFs) were generated using Open Drug Discovery Toolkit (ODDT) and Pybel, respectively, both OpenBabel dependent python modules [37–39]. Classification of the ETM-DB compounds into hierarchical taxonomic divisions was performed using ClassyFire, a web-based application for automated structural classification of chemical entities. ClassyFire’s chemical taxonomy system classifies compounds into kingdoms, superclasses, classes, subclasses, and more levels using computable structural rules [40]. BeautifulSoup, a Python library for pulling data out of HTML and XML file, was used to crawl the ClassyFire website and get each compound’s taxonomy, superclass and class.

#### Physicochemical, ADMET, and drug-likeness properties of Ethiopian herbal medicine compounds

PubChemPy was used to get predicted physicochemical properties of ETM-DB phytochemicals from the PubChem database. These properties include octanol/water partition coefficient (XLogP), topological polar surface area (TPSA), stereochemical complexity, formal charge of the compound, number of hydrogen bond donors and acceptors, number of rotatable bonds, number of stereo and heavy atoms, number of isotopes, number of stereocenters, and number of covalently-bonded units.

ADMET properties play key roles in the discovery/development of drugs [32]. ADMET related profiles for ETM-DB compounds were predicted using an admetSAR. We used BeautifulSoup python library to get predicted ADMET properties from admetSAR using compound SMILES as input. These properties include Human intestinal absorption (HIA), Blood-brain barrier (BBB) permeability, Caco-2 permeability, P-glycoprotein substrate, P-glycoprotein inhibitor I and II, renal organic cation transporter, CYP450 2C9 substrate, CYP450 2D6 substrate, CYP450 3A4 substrate, CYP450 1A2 inhibitor, CYP450 2C9 inhibitor, CYP450 2D6 inhibitor, CYP450 2C19 inhibitor, CYP450 3A4 inhibitor, CYP Inhibitory Promiscuity, human Ether-a-go-go-Related gene (hERG) inhibition, AMES toxicity, biodegradability, carcinogenicity, and rat acute toxicity. Therefore, data fields of each compound include general information (name, structures, synonyms, chemical superclass, formula, SMILES, InChI, InChIKey, PubChem ID, and ChemSpider ID) and predicted physicochemical and ADMET properties.

We used the FAF-Drugs4 [41] webserver to assess the drug-likeness of the phytochemicals. FAF-Drugs4 performs an input data curation that removes large molecules and compounds containing some types of inorganic atoms. We used OpenBabel logP computation program and drug-like soft in house and published physchem filters of the FAF-Drugs4 webservice. In addition, we filtered out undesirable substructures moieties and retrieved covalent inhibitors.

#### Human target gene/proteins and phenotypes

We used python script to obtain human target gene/proteins related to the ETM-DB compounds and phenotypes from the COCONUT database with reference to the original sources. The phenotypes in ETM-DB include phenotype symptoms treated by Ethiopian herbal medicine and phenotypes related to ETM-DB compounds.

### Database management

The ETM-DB was built using webserver Apache 2.2.15 and database server MySQL 5.6.25 to manage a relational database and store the information. Users can search for the main entities as well as the relationships using identifiers, names or other attributes provided by the search menu.

## Utility and discussion

### Web interface of the database and future plans

**Fig. 2:**
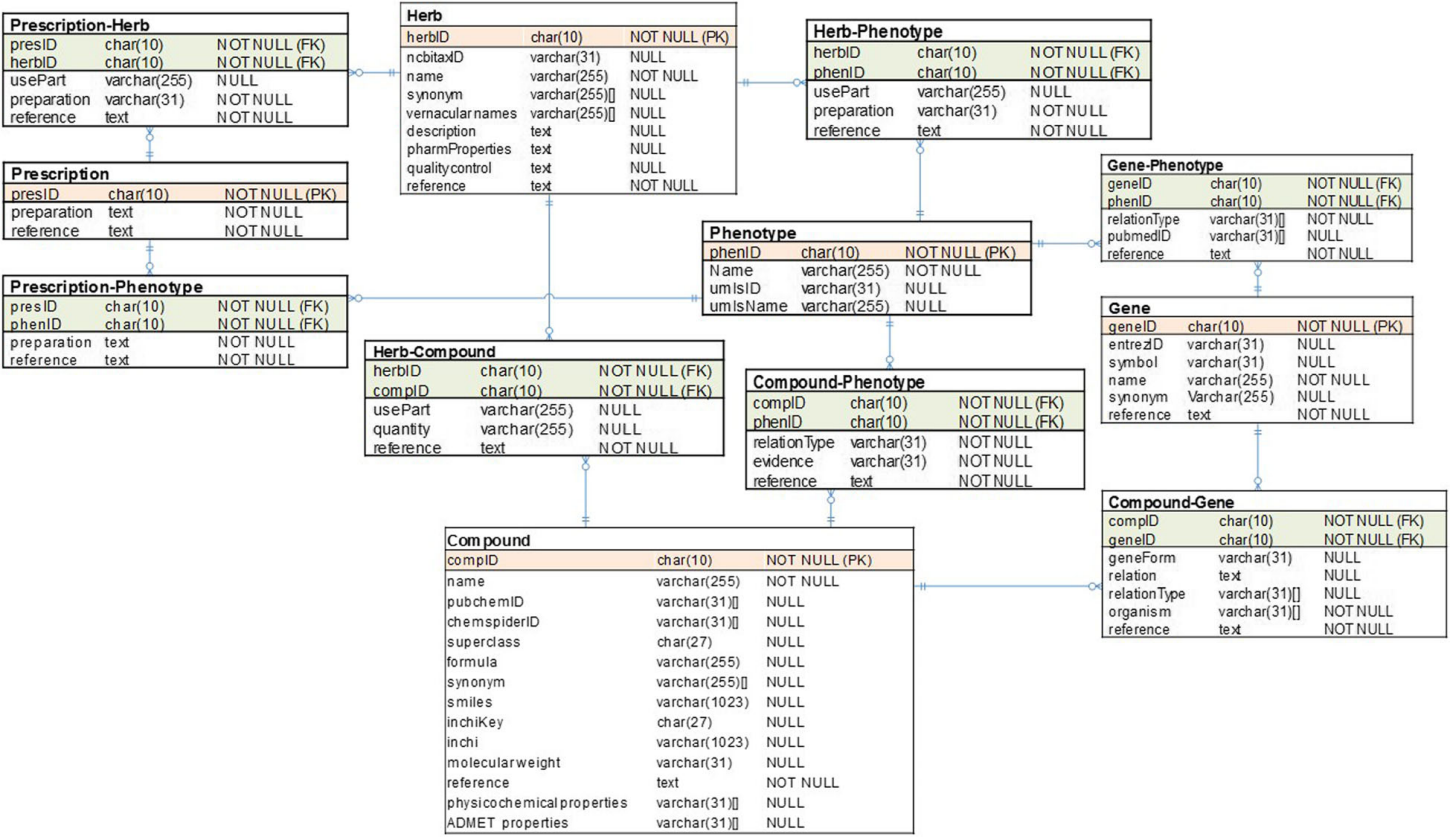
ETM-DB structure. The five entity tables are herb, prescription, compound, phenotype, and target gene/protein; and the seven relationships are herb-phenotype, prescription-herb, prescription-phenotype, herb-compound, compound-phenotype, compound-gene/protein, and phenotype-gene/protein

ETM-DB contains five entities and seven relationship tables. The main entities are herb, prescription, compound, phenotype, and gene/protein. The relationships between entities are herb-phenotype, prescription-herb, prescription-phenotype, herb-compound, compound-phenotype, compound-gene/protein, and phenotype-gene/protein. Information of the compounds in herbs and their targets is essential to predict its potential interaction and metabolism during drug development research. In developing countries such as Ethiopia, where synthetic chemistry is not well developed, knowledge of the active compounds and their target gene/protein is an important means to select, grow, and commercially extract the component(s) from locally available plants [42]. Furthermore, the herb-phenotype, prescription-herb, and prescription-phenotype relationships in ETM-DB can be valuable information for traditional medicine practitioners. Figure 2 depicts the structure of ETM-DB.

**Fig. 3:**
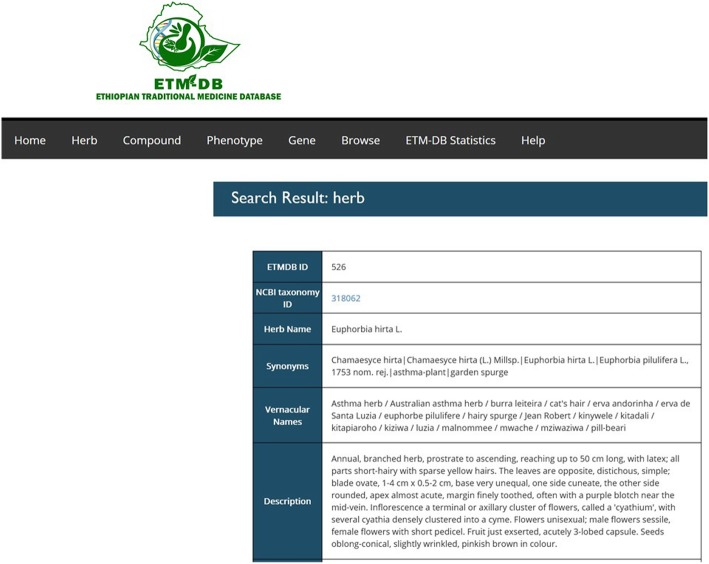
Web interface of ETM-DB. Snapshot of part of query results for a medicinal herb search

The ETM-DB website interface allows users to search for entities and their relationships using (a) name, ETM-DB ID, and NCBI taxonomy ID for herbs, (b) name, ETM-DB ID, InChI, InChIkey, SMILES, and formula for compounds. Users can also search compounds in ETM-DB directly by drawing the structure in the ‘Structure Search’ Field. The compound structure search incorporates JSME applet [43], which supports drawing and editing of molecules and reactions. (c) name, ETM-DB ID, UMLS name, and UMLS ID for phenotypes, and (d) name, ETM-DB ID, Entrez ID, and gene symbol for gene/proteins. Search results for entities and relationships including references to the sources are displayed in the web interface. The herbs search result page shows the herb attributes and related compounds, phenotypes, and prescriptions. The partial snapshot shown in Fig. 3 is a display for query result of a herb. Search results for a compound display compound attributes and related herbs, phenotypes, and gene/proteins. The compound attributes include name, structures, synonyms, chemical superclass, formula, SMILES, InChI, InChIKey, PubChem ID, ChemSpider ID, and predicted physicochemical and ADMET properties. The 3D structure can also be viewed by clicking on the link, which uses JSmol application [44]. Users can also download 2D and 3D structure of the compounds as SDF format. Phenotype search results display associated herbs, compounds, gene/proteins, and prescriptions. Similarly, gene/protein query results show gene/protein attributes, related compounds, and phenotypes. For each query result, users can click each entity identifier to view a different page including detailed data about the related entities.

ETM-DB would be more helpful if it includes more information such as quantitative composition and images of the herbs. Therefore, in the future updates, we will enrich ETM-DB with these and other details we might have missed. We believe that there are many Ethiopian herbs and related compounds we have not covered in this work. Most of the knowledge on Ethiopian traditional medicine is still mainly of an oral nature and thus not appropriately documented. Those that are documented in traditional literatures are scattered in different places within or outside the country owned by either individuals or institutions such as churches, monasteries, and libraries. In addition, Ethiopia is a country with more than 80 languages, which creates another barrier to access and document some information. Moreover, as there are not many laboratories and dedicated researchers, only a few species of Ethiopian traditional medicine herbs have been studied for their compound composition and pharmacological properties.

ETM-DB will be an ongoing work that needs continuous updates through further literature mining and manual curation. In addition to plants based natural products, we will consider including traditional medicines of animals and minerals origin. Collecting biological activity of the compounds is also part of our further plan. With enough collaboration and effort, ETM-DB can be scaled to include other African herbs. Furthermore, we will continue improving our webpage and webserver by incorporating various features deemed important.

## Summary of the content

**Fig. 4:**
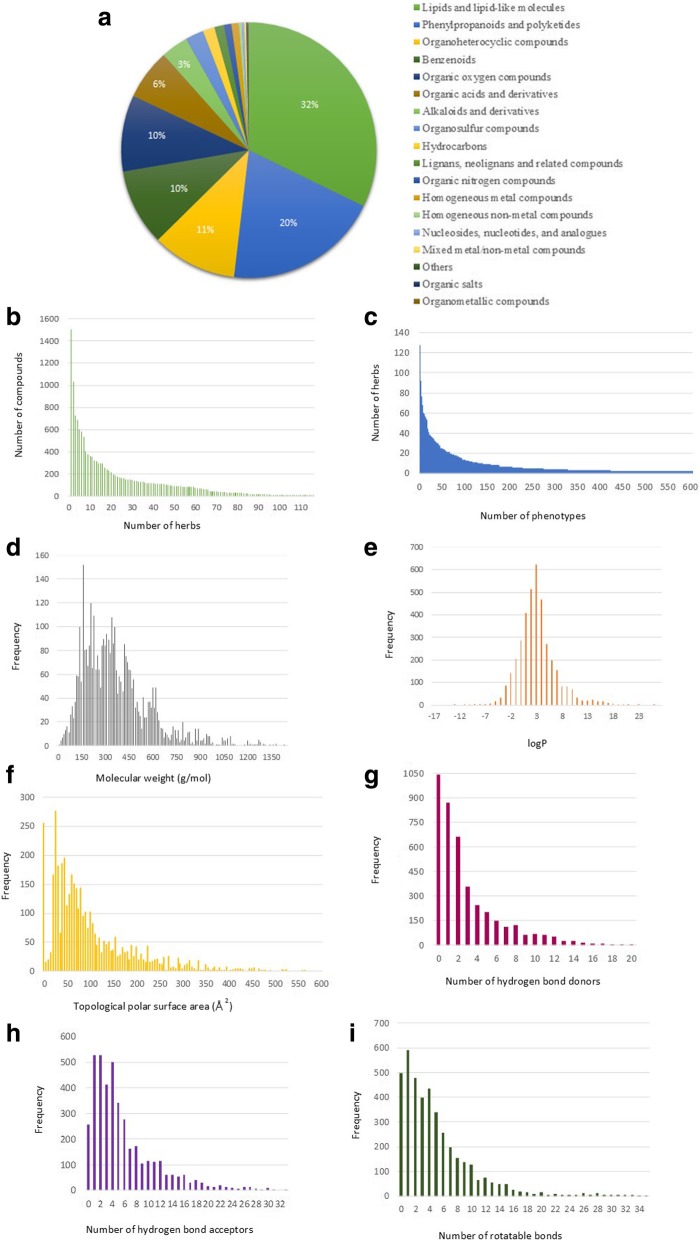
Basic statistics of ETM-DB. **a** Pie chart of distribution of the ETM-DB phytochemicals across different chemical superclasses obtained from ClassyFire. **b** Bar graph of the number of compounds per Ethiopian medicinal herbs. **c** Bar graph of the number of phenotypes per Ethiopian medicinal herbs. **d**-**i** Bar graphs of the molecular weight (g/mol), logP, TPSA (Å^2^), number of hydrogen bond donors, number of hydrogen bond acceptors, and number of rotatable bonds of compounds in ETM-DB

ETM-DB covers information on about 1054 Ethiopian medicinal herbs and 16,426 herb-compound relations. ETM-DB also has 162,632 relations between compounds and human target gene/proteins obtained from the COCONUT database with reference to the sources. We used ClassyFire webserver for chemical classification of 3930 ETM-DB compounds. The 3930 ETM-DB compounds are distributed across 22 superclasses and 200 classes of ClassyFire. Among the 22 superclasses, lipids and lipid-like molecules, phenylpropanoids and polyketides, and organoheterocyclic compounds are the top three superclasses with 1266, 772, and 423 compounds, respectively as shown in Fig. 4a. Among 200 chemical classes, prenol lipids, flavonoids, and organooxygen compounds, are the top three classes with 808, 413, and 379 compounds, respectively. ETM-DB includes records on 5779 herb-phenotype associations. In Fig. 4b, it is noted that 20 herbs contain more than 200 compounds and the remaining herbs have a considerable amount of compounds. This shows that Ethiopian herbs have a huge potential as a source of many compounds that can target a variety of diseases. Figure 4c shows a bar graph of the number of phenotypes per herb in ETM-DB. A majority of the 1054 herbs have less than 15 recorded traditional uses while a small amount of (about 70) herbs have more than 20 traditional uses. Among the 1054 Ethiopian medicinal herbs in our database, Croton macrostachyus Hochst. ex Delile, *Euphorbia hirta* L., and Vernonia amygdalina Del. have large number of recorded therapeutic uses (phenotypes) - 128, 92, and 90 respectively. Moreover, ETM-DB also contains data on 1879 prescription-herb relations, which comprises 573 multi-herb prescriptions from some combination of 265 Ethiopian medicinal herbs.

### Physicochemical, ADMET, and drug-likeness properties of Ethiopian herbal medicine compounds

Predicted physicochemical and ADMET properties of ETM-DB compounds are included in our database. We also assessed the drug-likeness of ETM-DB compounds using physicochemical properties computed through FAF-Drugs4 webserver. From the total 4285 ETM-DB compounds, 4080 of them passed the FAF-Drugs4 input curation stage. Then, we used OpenBabel logP computation program, and drug-like soft filtering values (molecular weight = 100–600 Da, log*P* = − 3 to 6, hydrogen bond acceptors ≤12, hydrogen bond donors ≤7, TPSA≤180 Å^2^, number of rotatable bonds ≤11, number of rigid bonds ≤30, size of rings ≤6, maximum size of rings ≤18, Carbons = 3–35, hetero atoms = 1–15, ratio of non-carbon atoms to carbon atoms = 0.1–1.1, charge of the compound ≤4), and filtered out undesirable substructures moieties as well as retrieved covalent inhibitors. This resulted in 876 compounds that have acceptable drug-likeness, 375 compounds with intermediate drug-likeness, and 2829 compounds with insufficient drug-likeness properties. Figure 4(d-i) shows the frequency distribution of some physicochemical properties of the compounds in ETM-DB.

### Comparison of ETM-DB with oriental and western small molecule databases

We compared the manually curated compounds found in Ethiopian traditional medicinal herbs with oriental medicine compounds and western medicine small molecule compounds. By comparing the 453 manually curated ETM-DB compounds with a set of 43,415 TCMID, 25,050 TCM-ID, 10,033 KTKP [45], 712 SANCDB, and 8578 DrugBank [46] compounds, 225, 135, 105, 9, and 37 of the compounds respectively were found in common. After removing redundancies, we found that 206 compounds are unique to ETM-DB compared to the above databases. Thus, compounds from Ethiopian traditional medicine, in addition to oriental medicine, may offer an extensive opportunity for novel drug discovery.

**Fig. 5:**
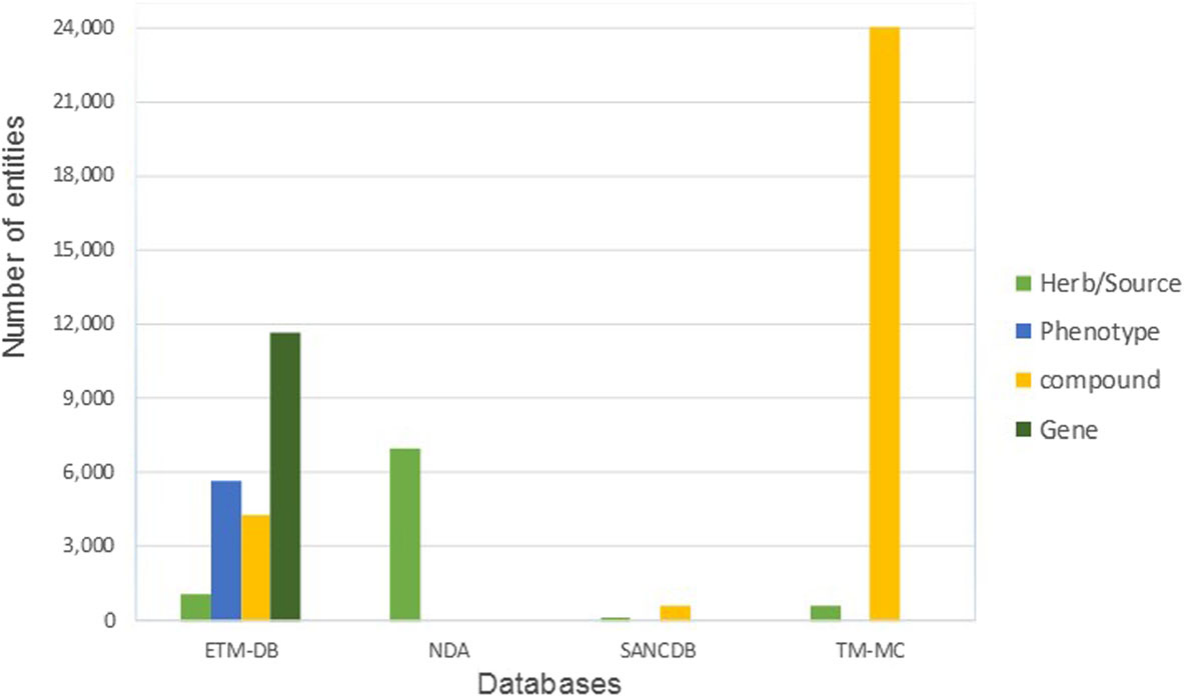
Bar graph of comparison of the amount of ETM-DB main entities with related natural product databases

Table 1 and Fig. 5 show the types and quantities of ETM-DB entities alongside that of related natural product databases (NDA, SANCDB, and TM-MC). Most of the related natural product databases have limited, scattered, and incomplete information. Furthermore, most of the African natural product databases focus on specific entities such as herbs or compounds. This shows that our database is more comprehensive regarding types and amount of entities.

**Table 1:** Comparison of main entities, relationships, and other features in ETM-DB with related natural product databases

Databases	NDA	SANCDB	TM-MC	ETM-DB
Main entities				
Herb	7000	143	602	1054
Compound	No	600	24,018	4285
Phenotype	No	No	No	5621
Prescription	No	No	No	573
Gene/protein	No	No	No	11,621
Relationships				
Herb-Phenotype	No	No	284	5779
Herb-Compound	No	600	40,405	16,426
Prescription-Herb	No	No	No	1879
Prescription-Phenotype	No	No	No	573
Compound-Phenotype	No	284	No	105,202
Compound-Gene/protein	No	No	No	162,632
Phenotype-Gene/protein	No	No	No	16,584
Other Features				
Publicity	Yes	Yes	Yes	Yes
Chemical classification	No	Yes	No	Yes
Downloadable structure	No	Yes	Yes	Yes
Physicochemical properties	No	Yes	No	Yes
ADMET properties	No	No	No	Yes

## Conclusion

Natural products have been utilized as traditional medicine since ancient times. It is evident that many modern drugs have been derived or inspired from NPs [47]. Several natural product databases were developed to assist large-scale screening of natural products for drug development. In this work, we have developed ETM-DB, an integrated relational database of 1054 Ethiopian traditional medicinal herbs with 1463 traditional therapeutic uses and 4285 related compounds. ETM-DB provides interrelationships among these entities (herbs, prescriptions, phenotypes, compounds, and gene/proteins) with references to the original sources. It also contains chemical classification, predicted physicochemical and ADMET properties of the compounds, which were obtained using various cheminformatics tools. We have also evaluated drug-likeness of the compounds using FAF-Drugs4 webserver and found that 876 compounds have acceptable drug-likeness properties. This indicates the immense potential of Ethiopian herbs for drug discovery and development.

ETM-DB is the largest, freely available web-based resource for Ethiopian natural products to date. Users can search a specific entity; find corresponding attributes and its relationships with other entities with references. As we have organized various entities and their descriptive properties alongside their interrelationships, which are not usually found consolidated into one database, we believe that researchers interested in utilizing Ethiopian natural products for drug discovery process can benefit a lot from this database.

We intend to continue enriching and expanding our database by mining and analyzing further relevant sources. In addition to Ethiopian herbs, this database can easily be expanded to include other African herbs. We also have a plan to improve our webserver by incorporating various features. It is also important to include quantitative compositions and the biological activity of each herb and herb parts, as it is valuable for assessing and developing medicine formulations.

## Data Availability

The current version of ETM-DB datasets is openly accessible at http://biosoft.kaist.ac.kr/etm. Compact format of the datasets used for analysis purposes such as drug-likeness evaluation during the current study are available from the corresponding author on a reasonable request.

## Abbreviations

2D: two-dimensional
3D: three-dimensional
ADMET: absorption, distribution, metabolism, excretion, and toxicity
admetSAR: ADMET structure-activity relationship
AfroDB: a Select Highly Potent and Diverse Natural Product Library from African Medicinal Plants
BBB: Blood-brain barrier
BFN: Bio-Food Network
CamMedNP: Building the Cameroonian 3D structural natural products database for virtual screening
CEMTDD: Chinese Ethnic Minority Traditional Drug Database
COCONUT: Compound Combination-Oriented Natural Product Database with Unified Terminology
ConMedNP: A natural product library from Central African medicinal plants for drug discovery
CTD: Comparative Toxicogenomics Database
DCDB: Drug Combination Database
ETM-DB: Integrated Ethiopian Traditional Herbal Medicine and Phytochemicals Database
FAF-Drugs4: free ADME-tox filtering computations for chemical biology and early stages drug discovery
FDA: US Food and Drug Administration
FooDB: The Food Database
HIA: Human intestinal absorption
hERG: a human Ether-a-go-go-Related Gene
ID: Identifier
IMPPAT: A curated database of Indian Medicinal Plants, Phytochemistry And Therapeutics
InChI: International Chemical Identifier
JSME: a free molecule editor in JavaScript
JSmol: an open source molecule viewer in JavaScript
KNApSAcK: A Comprehensive Species-Metabolite Relationship Database
KTKP: Korean Traditional Knowledge Portal
MATADOR: Manually Annotated Targets and Drugs Online Resource
NANPDB: A Resource for Natural Products from Northern African Sources
NAPRALERT: Natural Products Alert
NCBI: National Center for Biotechnology Information
NDA: Natural Products Database for Africa
NER: Named Entity Recognition
NP: Natural product
ODDT: Open Drug Discovery Toolkit
p-ANAPL: Pan-African Natural Products Library
SANCDB: South African natural compound database
SDF: Structure Data File
SIDER: Side Effect Resource
SMILES: Simplified Molecular Input Line Entry System
STITCH: Search tool for interactions of chemicals
TCAM: traditional, complementary and alternative medicine
TCM: Traditional Chinese Medicine
TCM-ID: Traditional Chinese Medicine Information Database
TCMID: Traditional Chinese Medicine Integrative Database
TM-MC: A database of medicinal materials and chemical compounds in Northeast Asian traditional medicine
TPSA: Topological polar surface area
TTD: Therapeutic Target Database
UMLS: Unified Medical Language System
XLogP: Octanol/Water partition coefficient
